# Development of Latex Microsphere-Based Immunochromatographic Strips for Detecting Key Aflatoxins

**DOI:** 10.3390/toxins17090426

**Published:** 2025-08-22

**Authors:** Jie Wang, Wangzhuo Fu, Xuezhen Ma, Lin Chen, Weitao Song, Sumei Ling, Hongyun Qian, Shihua Wang, Zhenhong Zhuang

**Affiliations:** 1 Key Laboratory of Pathogenic Fungi and Mycotoxins of Fujian Province, Key Laboratory of Biopesticide and Chemical Biology of Education Ministry, Proteomic Research Center, School of Life Sciences, Fujian Agriculture and Forestry University, Fuzhou 350002, China; wangjie10909@163.com (J.W.); fuwangzhuo@foxmail.com (W.F.); mapha@fafu.edu.cn (X.M.); m18705194907_1@163.com (L.C.); xsm19980225@163.com (W.S.); lsmpu2008@163.com (S.L.); 2 Fujian Baimeng Medical Technology Company Limited, Fuzhou 350000, China; qhy6674@163.com

**Keywords:** hybridoma, AFB_1_, AFG_1_, latex microsphere, immunochromatographic test strip

## Abstract

Due to the severe hazard of aflatoxins (AFs) to humans, it is of great significance to detect the key aflatoxins, aflatoxin B_1_ (AFB_1_) and aflatoxin G_1_ (AFG_1_), in food and feed in simple, rapid, and semi-quantitative ways. The hybridoma clone 3A1 was prepared in this study, and anti-AFB_1_ monoclonal antibody (mAb) with high specificity and affinity (9.38 × 10^8^ L/mol) from 3A1 was purified. The indirect competitive enzyme-linked immunosorbent assay (ic-ELISA) demonstrated that the linear detection range for AFB_1_ was 0.029–1.526 ng/mL with a limits of determination (LOD) of 0.023 ng/mL. A latex microsphere-based immunochromatographic test strip (LM-ICTS) was constructed based on 3A1, which showed that the strip could detect AFB_1_ (LOD: lower than 1.79 ng/mL) and AFG_1_ (LOD: lower than 8.08 ng/mL), and the linear detection ranges for AFB_1_ and AFG_1_ are 1.79–48.46 ng/mL and 8.08–107.40 ng/mL, respectively. The average recoveries of intra-assay and inter-assay for peanuts were (98.4 ± 4.7)% and (92.6 ± 7.6)%, and the average coefficient of variation (CVs) were 4.38% and 8.15%, respectively. For sunflower seeds, the intra-assay and inter-assay recoveries were (94.4 ± 7.2)% and (89.2 ± 4.3)%, and the average CVs were 6.6% and 4.9%, respectively. In summary, the developed LM-ICTS exhibited excellent sensitivity and specificity, which provided a rapidly stable on-site detection choice for AFB_1_ and AFG_1_ to contaminated agricultural samples, including grain and feed.

## 1. Introduction

The notorious fame of aflatoxins (AFs) is well known after they caused “Turkey-X” disease in England in 1960, which resulted in the death of over 100,000 turkeys [1]. As a cluster of mycotoxins, AFs are mainly produced by *Aspergillus flavus* and *A. parastiticus* on various important food and feed crops (including corn, peanuts, and cassava) during plant growth, harvest, storage, and processing in a wide region around the world between 40° N and 40° S latitudes [2]. The low molecular weight AFs are thermal stable (melting point: 237–309 °C) and hazardous chemical compounds, which mainly include aflatoxin B_1_ (AFB_1_, MW: 312), aflatoxin B_2_ (AFB_2_, MW: 314), aflatoxin G_1_ (AFG_1_, MW: 328), and aflatoxin G_2_ (AFG_2_, MW: 330) [3]. AFs are known to be strongly hepatotoxic, carcinogenic, and mutagenic to human and animals [4]. Therefore, the U.S. Food and Drug Administration (FDA) limits the total level of AFs in foods or feeds to 20 ppb [5].

Among AFs, AFB_1_ is the most toxic with a strong affinity for the liver [6]. In the liver, AFB_1_ is metabolized by the P450 enzyme, causing DNA damage and potentially leading to cell mutation and carcinogenesis [7]. AFG_1_ is the second most toxic type of AF containing a double furan ring and a lactone ring, which assigns it the characteristics of high toxicity [8,9]. Similar to AFB_1_, AFG_1_ tends to accumulate in the liver, causing liver damage and carcinogenesis [10]. Therefore, the development of rapid, accurate, and sensitive detection methods for AFB_1_ and AFG_1_ is of great significance for ensuring food safety and public health.

Many accurate and sensitive chromatography- and immunology-based detection methods for aflatoxins have been developed. Liquid chromatography (LC) coupled with a tandem MS (MS/MS) have been recently used to effectively validate mycotoxins, including AFs in maize and peanuts [11,12]. Enzyme-linked immunosorbent assay (ELISA) methods have been widely used for AFB_1_ detection in corn, raw peanuts, and peanut butter [13]. High performance liquid chromatography coupled with fluorescence detector (HPLC-FL) is used for the quantitative study of AFB_1_ from cereal samples with the limits of determination (LOD) of 0.002 μg/kg [14]. Aptarmer combined with lateral flow strips was designed to detect AFB_1_ in a dual-competitive approach within the linear range 0.1–1000 ng/mL in 20 min [15]. An AFB_1_ immunoassay detection method based on silica-encapsulated hollow gold nanoparticles (SEHGNs) was reported in 2015, by which the limit of detection of AFB_1_ was 0.1 ng/mL in about 30 min [16]. A strip based on gold immunochromatographic assay was developed, which could detect total AFs with LOD of 0.01 ng/mL [17]. It can be seen that most developed AF detection methods are focusing on AFB_1_ or total AFs. Reports showed that AFB_1_ is the most abundant AFs, usually accounting for more than 50% of total AFs, while AFG_1_ is the second most abundant, accounting for more than 35% [18,19]. In view of the different hazard extent and natural yield of AFs [3,20], there is an urgent need to develop a sensitive method to rapidly detect these key natural AFs (including AFB_1_ and AFG_1_) simultaneously.

In this study, a hybridoma clone 3A1 secreting a highly specific monoclonal antibody (mAb) against AFs was prepared. Then, the rapid detection methods based on latex microspheres (LM) and hybridoma clone 3A1 were established for AFB_1_ and AFG_1_ assay. This study provided a sensitive and rapid on-site semi-quantitative monitoring choice for AFB_1_ and AFG_1_ from contaminated grain, feed, and other agricultural samples in food and feed industries.

## 2. Results and Discussion

### 2.1. Preparationand Identification of Complete Antigen

**Figure 1:**
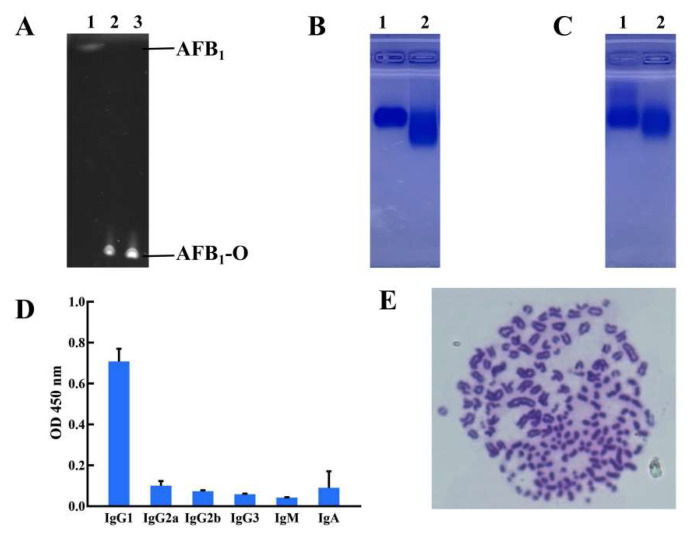
Complete antigen preparation and hybridoma screening. (**A**) The TLC analysis of AFB_1_ oximation product. Lane 1, AFB_1_. Lanes 2 and 3, AFB_1_-O. (**B**) The result of agarose gel electrophoresis for AFB_1_-BSA. Lane 1, BSA. Line 2, AFB_1_-BSA. (**C**) The agarose gel electrophoresis result for AFB_1_-HSA. Lane 1, HSA. Line 2, AFB_1_-HSA. (**D**) The subtype analysis of the hybridoma 3A1. (**E**) The chromosome number of the positive hybridoma 3A1.

To prepare the complete antigen of AFB_1_, AFB_1_ was oximated by carboxymethoxylamine hemihydrochloride (CMO), and analyzed by thin layer chromatography (TLC). As shown in Figure 1A, the oximation product of AFB_1_ (AFB_1_-O) is located below the AFB_1_ band, likely due to its higher polarity resulting in a slower migration rate on the TLC plate. Then, the AFB_1_-O was fixed on the carrier protein bovine serum albumin (BSA) and human serum albumin (HSA) by the method of 1-(3-Dimethylaminopropyl)-3-ethylcarbodiimide hydrochloride (EDC), and the results showed that the AFB_1_-BSA and AFB_1_-HSA in Lane 2 migrated faster than the corresponding standard in Lane 1 (Figure 1B,C). This is due to the change in the surface charge of the carrier protein, which alters the migration speed during electrophoresis. The results confirmed that the conjugates AFB_1_-BSA and AFB_1_-HSA were successfully synthesized. Similar to other mycotoxins, AFs are haptens with very low molecular weight (MW: about 312 to 330). Being haptens, AFs have no immunogenicity to stimulate the immune system to produce corresponding antibodies. To prepare complete antigens of AFB_1_, the study adopted a two steps method. Firstly, AFB_1_ was oximated by CMO. Secondly, under the activation of EDC, the complete antigen of AFB_1_ was formed by the connection of the “CO” chemical group in the oximation production (AFB_1_-O) with the “NH_2_” chemical group in carrier proteins through the formation of “C-N” chemical bond. The usual candidates of carrier proteins are BSA (67 kDa), HSA (66 kDa), keyhole limpet hemocyanin (KLH, 360 kDa), and ovalbumin (OVA, 43 kDa), etc. Among them, BSA has a number of lysine residues, and these residues are ideal positions to combine with haptens [21]. HSA also contains 59 lysine residues [22]. So BSA and HSA were chosen to be the carriers for the AFB_1_ haptens in the study.

### 2.2. Screening of Positive Hybridoma Cell

After six times of immunization with complete antigen AFB_1_-HSA, the titer of antiserum was detected by indirect ELISA (iELISA) with AFB_1_-BSA as the coated antigen. In the assay, the antiserum was diluted at 500, 1000, 2000, 4000, 8000, 16,000, 32,000, and 64,000 times, and the results showed that the OD_450 nm_ of the serum was beyond 1 after 32,000 times dilution, which reflected that the titers of the serum from both mice are all higher than 32,000. Then, mouse 1 with higher titer was selected to prepare the hybridoma cell by fusion of SP2/0 myeloma cells with the splenocytes under 50% PEG. The positive hybridoma clone 3A1 was selected for further limiting dilution and screened out by iELISA. The isotype of the mAb is important for monoclonal antibodies, so the isotype of hybridoma clone 3A1 was assayed with the Mouse Monoclonal Antibody Subtyping Kit (Solarbio in Beijing). The result reflected that the isotype of hybridoma 3A1 is IgG1 as shown in Figure 1D. The isotype of mAb is concerned with the characteristics of protein A or G binding, complement binding, kinetics of digestion, affinity, and so on [23]. IgG1 could be easily eluted with protein G or under low pH value by protein A-Sepharose [24,25]. IgG1 always shows higher affinity against target antigens compared to other isotypes [26,27]. The chromosome number of the positive hybridoma was calculated under inverted microscope, and the results showed that the chromosome number of the positive hybridoma 3A1 is 106 ± 3 (Figure 1E), which is consistent with the theoretical value, indicating that the positive hybridoma 3A1 was really the fusion of the SP2/0 myeloma cells with the splenocytes [28].

### 2.3. Characterization of the mAb from 3A1

**Figure 2:**
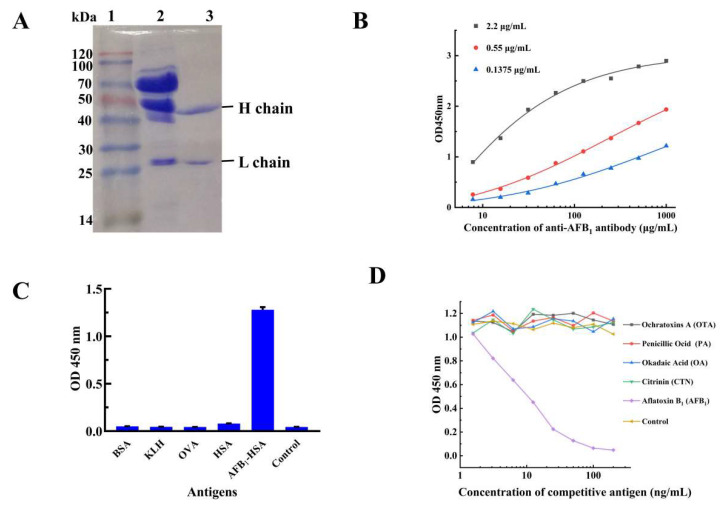
Characterization of the mAb from 3A1. (**A**) SDS-PAGE result of the purified anti-AFB_1_ mAb. Line 1, protein ladder. Line 2, the total proteins from the ascites. Line 3, the purified anti-AFB_1_ mAb from the ascites. (**B**) Affinity analysis of the anti-AFB_1_ mAb by iELISA (coated with 2.2 μg/mL, 0.55 μg/mL, 0.1375 μg/mL AFB_1_-BSA). (**C**) The specificity of the anti-AFB_1_ mAb to different carrier proteins analyzed by iELISA. (**D**) The specificity of the mAb to different small molecular mycotoxins analyzed by icELISA.

The ascites was prepared by the intraperitoneal injection of the paraffin oil primed Balb/c mice with the positive hybridoma 3A1. Then, the mAb was purified with protein G, and analyzed with Sodium dodecyl sulfate-Polyacrylamide gel electrophoresis (SDS-PAGE). The results showed that the mAb was composed of a pair of heavy chains (about 50 kDa) and a pair of light chains (about 25 kDa), while no other protein bands could be found in Lane 2, indicating that the mAb was successfully purified (Figure 2A). The affinity of mAb from hybridoma 3A1 was assessed by iELISA, and the result revealed that the affinity constant (*Kaff*) of this mAb is 9.38 × 10^8^ L/mol (Figure 2B), which meant that the mAb from hybridoma 3A1 is a high affinity antibody. The affinity constant of mAb from 3A1 is higher than the *Kaff* (2.81 × 10^8^ L/mol) of the anti-AFB_1_ mAb prepared by Jiang et al. (2020) [29]. The specificity of the mAb was determined by iELISA and indirect competed ELISA (icELISA), and the result of iELISA showed that there was no cross reactivity to other carrier antigens, including KLH, OVA, BSA, and HSA (Figure 2C). The result of icELISA also reflected that, except for AFB_1_, no other low molecular weight antigen, including ochratoxins A (OTA), penicillic ocid (PA), okadaic acid (OA), and citrinin (CTN), could rival AFB_1_ to bind the mAb (Figure 2D). The above results indicated that this high affinity and specific mAb is available for future use in highly effective aflatoxin detection.

### 2.4. Development of icELISA for Aflatoxin Detection

**Figure 3:**
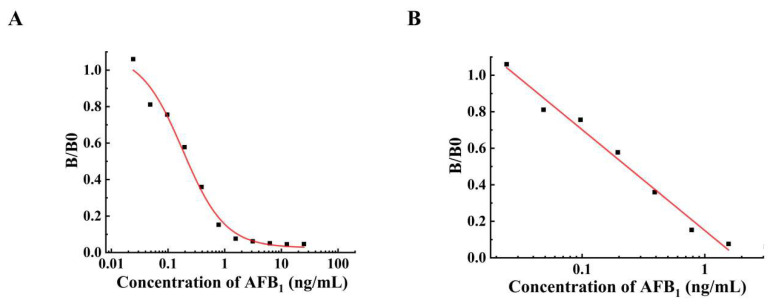
The standard curves for AFB_1_ detection. (**A**) The typical calibration curve deduced from the plotting (B/B0) against AFB_1_ concentration. The data gained with and without competition factor in various concentrations are referred to as B and B0, respectively. (**B**) The linear part of the standard curve. The linear equation is y = 1.1205 − 0.55242x, R^2^ = 0.981.

Seeing that the mAb from hybridoma 3A1 has high affinity and specificity, the icELISA was applied to set up a standard curve for aflatoxin detection. The equation of logistic curve (y = 0.0259 + (1.09243 − 0.0259)/(1 + (x/0.18308)^0.96476^) was deduced from the plotting (B/B0) against AFB_1_ concentration in the typical calibration curve (Figure 3A), with a correlation coefficient (adjusted R-Square) of 0.982. The linear equation deduced from Figure 3B is y = 1.1205 − 0.55242x with an adjusted R-Square (R^2^) of 0.981. The half inhibitory concentration (IC50) of AFB_1_ was 0.2203 ng/mL, and the linear range of detection was 0.029–1.526 ng/mL with a lower detection limit (LOD) of 0.023 ng/mL AFB_1_. The LOD of the mAb in this study is obviously lower than the previously reported 4 μg/kg by Lipigorngoson et al. (2003) [30], 0.128 reported by Chu et al. (2015) [31], and 0.15 μg/kg by Jiang et al. (2020) [29], respectively. To determine the recovery of the mAb, the final concentration of AFB_1_ was added into peanut before extraction at 2, 1, 0.5, and 0.25 μg/kg, respectively. The concentration of AFB_1_ in the extract was determined by icELISA method, and three samples in each group were analyzed in parallel. As shown in Table 1, the average recoveries in and out of batches were (90.6 ± 3.4)% and (88.9 ± 2.2)%, respectively, and the coefficients of variation (CV) were 3.8% and 2.4%, respectively.

### 2.5. The Preparation of the Latex Microspheres (LM) Probe

**Figure 4:**
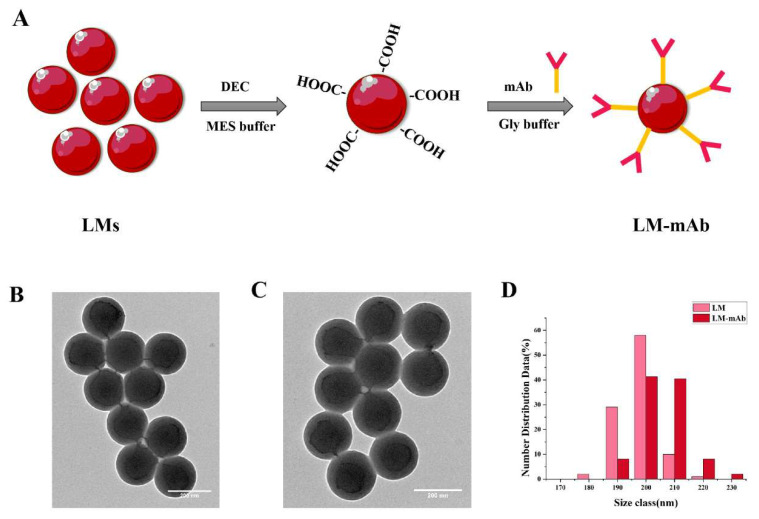
The preparation of LM-mAb. (**A**) The schematic diagrams of the preparation of the LM probe. (**B**) TEM of naked latex microspheres. (**C**) TEM of LM-mAb. (**D**) The particle size before and after LM coupling with mAb.

LM not only has a large specific surface area and rich colors, but also possesses excellent stability compared to colloidal gold and nanoflower [32], so it has been widely used as a reporting label in the fields of food safety monitoring and clinical diagnosis [33,34]. In this study, LM was used as a tag conjugated with the mAb to prepare the LM chromatographic strip for the rapid detection of AFB_1_ and AFG_1_. The schematic diagrams of the preparation of LM and the LM probe are shown in the Figure 4A. The red LM particles were firstly carboxylated with EDC and then used to label the mAb. As shown in the Figure 4B, the transmission electron microscope (TEM) images revealed that the surface of the untreated LM is smooth and uniformly dispersed in water solution. In contrast, the mAb-coated LM exhibited a rougher surface with a noticeable enlargement in particle size compared to the above naked LM (Figure 4C,D). After the conjugation of the mAb, the average particle size was increased by 10 nm (the average particle size of LM was 197.780 ± 0.0332 nm, and LM-mAb was 208.292 ± 0.1464 nm). This reflected that LM particles were effectively coated with mAb in the mAb-coated LM group.

### 2.6. Construction of Latex Microsphere-Based Immunochromatographic Test Strip

**Figure 5:**
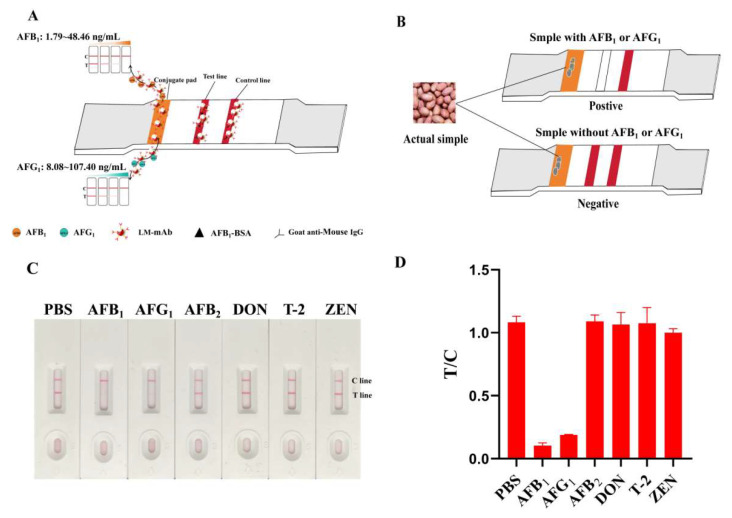
Construction of LM-ICTS. (**A**) Schematic diagram of LM-ICTS for AF detection using LM-3A1 probe as trace label. (**B**) The model of application of LM-ICTS in real samples. (**C**) The specificity analysis of the prepared LM-ICTS. (**D**) The value of T/C of LM-ICTS for the specificity analysis in the Panel (**C**).

Subsequently, the latex microsphere-based immunochromatographic test strip (LM-ICTS) was assembled (Figure 5A) using the aforementioned LM-mAb as the detection probe, and then it was applied for the AFs detection from actual samples (Figure 5B). The probes were collected and the experimental conditions, such as the concentrations of goat anti-mouse IgG, AFB_1_-BSA, and LM-mAb, were optimized. Under the optimal experimental conditions, the concentration of goat anti-mouse IgG was 0.25 mg/mL, the concentration of AFB_1_-BSA was 137.86 μg/mL, and the volume of LM-mAb was 3 μL. The specificity of the LM-ICTS was tested with 100 ng/mL zearalenone (ZEN), deoxynivalenol (DON), and T-2 toxin as antigens (each 100 µL). AFG_2_ was produced by few *A. flavus* or *A. parastiticus* strains, and its toxicity and yield was lower compared to AFB_1_, AFB_2,_ and AFG_1_ [35,36,37]; therefore, AFB_1_, AFB_2,_ and AFG_1_ were chosen for the specificity assay with the same volume and concentration. The result showed that the red color on the T lines for AFB_1_ and AFG_1_ disappeared, while there was no cross-reactivity with the other micromolecular toxins (Figure 5C,D). This indicated that the prepared monoclonal antibody has good specificity for AFB_1_ and AFG_1_ in the LM-ICTS.

**Figure 6:**
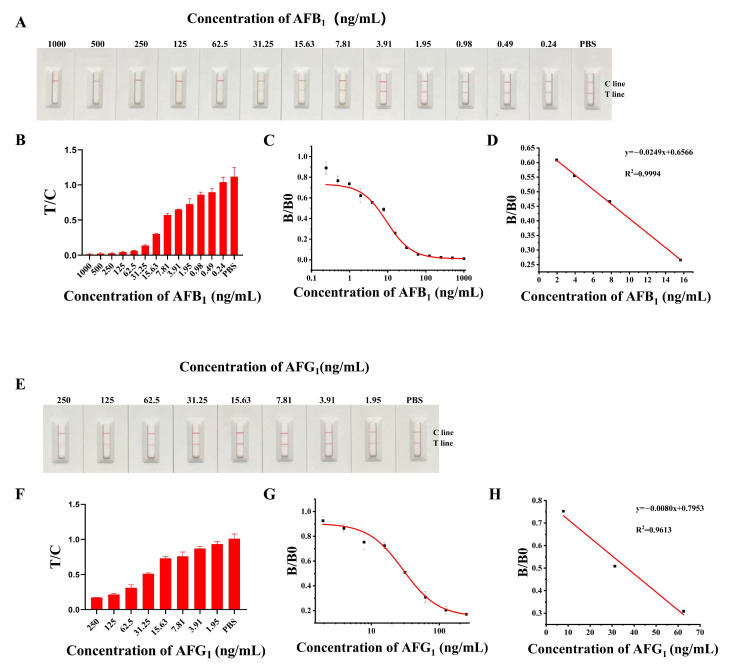
The sensitivity analysis of LM-ICTS. (**A**) The analysis of the sensitivity of LM-ICTS for AFB_1_. (**B**) The value of T/C of LM-ICTS for AFB_1_ sensitivity based on Panel (**A**). (**C**) The typical calibration curve deduced from the plotting (B/B0) against AFB_1_ concentration. (**D**) The standard curve of LM-ICTS for AFB_1_ detection (y = −0.0249x + 0.6566, R^2^ = 0.999). (**E**) The analysis of the sensitivity of LM-ICTS for AFG_1_. (**F**) The value of T/C of LM-ICTS for AFG_1_ sensitivity based on Panel (**E**). (**G**) The typical calibration curve deduced from the plotting (B/B0) against AFG_1_ concentration. (**H**) The standard curve of LM-ICTS for AFG_1_ detection (y = −0.0080x + 0.7953, R^2^ = 0.961).

Based on the above results, the sensitivity of the LM-ICTS was further evaluated using AFB_1_ and AFG_1_ with a series of concentrations ranging from 0 to 1000 ng/mL. It was clearly demonstrated that with the concentrations of AFB_1_ and AFG_1_ increased, the red T line gradually became weaker (Figure 6A,E) and the T/C ratio also decreases with the increased concentration of AFB_1_ and AFG_1_ (Figure 6B,F). Finally, the results showed that the limit of detection (LOD) of the prepared LM-ICTS for AFB_1_ is 1.79 ng/mL, and for AFG_1_ is 8.08 ng/mL (Figure 6C,G), with a linear detection range of 1.79–48.46 ng/mL for AFB_1_ and 8.08–107.40 ng/mL for AFG_1_ (Figure 6D,H). Therefore, the LOD for AFB_1_ was lower than the maximum residue limits specified by China (GB 2761-2017) for AFB_1_ in foodstuffs (20 µg/kg for peanuts, 20 µg/kg for corn, and 5 µg/kg for other cereals) [38,39]. Likewise, the LOD for AFG_1_ of this LM-ICTS is also lower than the regulatory limit specified by that standard (10 µg/kg for corn, 50 µg/kg for compound feed, and 100 µg/kg for concentrated feed). It means that the LM-ICTS constructed in this study is competent for the application in AFB_1_ and AFG_1_ on-site detection.

### 2.7. Analysis of Spiked Samples

**Figure 7:**
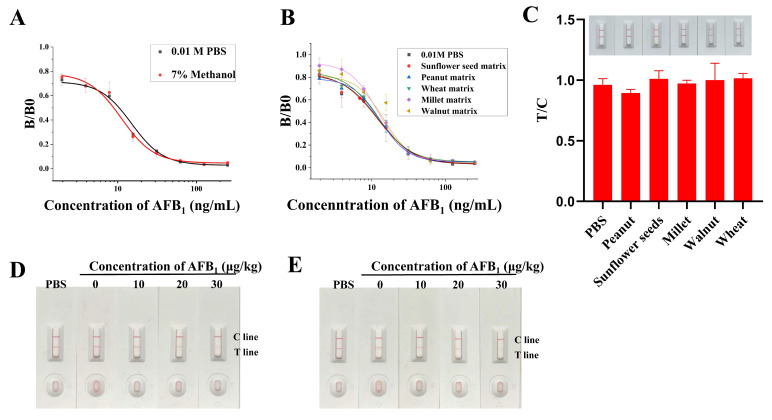
The application of the LM-ICTS in real samples analysis. (**A**) Comparison of the sensitivity of LM-ICTS to AFB_1_ under the solvent of methanol and PBS. Red curve, LM-ICTS detected AFB_1_ which was diluted by 7% methanol into 250, 125, 62.5, 31.25, 15.63, 7.81, 3.91, and 1.95 ng/mL. Black curve, AFB_1_ was diluted by PBS into 250, 125, 62.5, 31.25, 15.63, 7.81, 3.91 and 1.95 ng/mL. (**B**) Matrix effects were evaluated by food sample spiking. Black curve, LM-ICTS detected AFB_1_ which was diluted by PBS into 250, 125, 62.5, 31.25, 15.63, 7.81, 3.91, and 1.95 ng/mL. Red curve, LM-ICTS detected AFB_1_ which was diluted by sunflower seed extract (1:9 dilution) into 250, 125, 62.5, 31.25, 15.63, 7.81, 3.91, and 1.95 ng/mL. Blue curve, LM-ICTS detected AFB_1_ which was diluted by peanut extract (1:9 dilution) into 250, 125, 62.5, 31.25, 15.63, 7.81, 3.91, and 1.95 ng/mL. Green curve, LM-ICTS detected AFB_1_ which was diluted by wheat extract (1:9 dilution) into 250, 125, 62.5, 31.25, 15.63, 7.81, 3.91, and 1.95 ng/mL. Purple curve, LM-ICTS detected AFB_1_ which was diluted by millet extract (1:9 dilution) into 250, 125, 62.5, 31.25, 15.63, 7.81, 3.91, and 1.95 ng/mL. Yellow curve, AFB_1_ was diluted by walnut extract (1:19 dilution) into 250, 125, 62.5, 31.25, 15.63, 7.81, 3.91 and 1.95 ng/mL. (**C**) Detection of real samples from supermarkets. (**D**) Peanuts were spiked with AFB_1_ in different concentrations. (**E**) Spiked AFB_1_ at different concentrations in sunflower seeds.

Randomly purchased peanuts, wheat, sunflower seeds, and other foods from supermarkets were used to conduct detection and recovery experiments to evaluate the reliability of the developed LM-ICTS. The testing samples extracted from the above foods were dissolved in 7% methanol, and the effect of 7% methanol on the sensitivity of the LM-ICTS was evaluated by comparing the effect of the methanol solvent with PBS. The result showed that 7% methanol does not significantly affect the sensitivity of LM-ICTS, as shown in Figure 7A. For evaluating the accuracy of the LM-ICTS under complicated food matrixes, the above sample extracts spiked with known concentrations (at a concentration gradient of 250, 125, 62.5, 31.25, 15.63, 7.81, 3.91, and 1.95 ng/mL) of AFB_1_ were analyzed using LM-ICTS. The result confirmed that matrixes in the food sample extracts had little effect on LM-ITCS (Figure 7B). Therefore, the LM-ICTS can be used in the following food sample testing.

The results of following actual sample testing showed that two bands clearly appeared in each actual sample, which meant that the actual samples were not contaminated by aflatoxins (Figure 7C). For further assessing the reliability of the LM-ICTS in actual sample detection, the spiking food samples were analyzed. The results showed that as the concentration of AFB_1_ in the samples increases, the T line gradually fades (Figure 7D,E), indicating that LM-ITCS is capable of detecting AFB_1_ in real samples. In addition, recovery rates assay was carried out for this LM-ICTS. The inter-assay and intra-assay average recovery rates for peanuts were 98.4% and 92.6%, with a CV of 4.38% and 8.15%, respectively. In the sunflower seeds, the inter-assay and intra-assay average recovery rates were 94.4% and 89.2%, and the corresponding CV was 6.64% and 4.87%, respectively (Table 2). The results demonstrated that the CV of intra- and inter-assay was less than 10%. The low CV and reliable recoveries confirmed that the LM-ICTS developed in this study is a competent candidate for rapid and sensitive determination of trace AFB_1_ and AFG_1_ residues in agricultural products.

## 3. Conclusions

The hybridoma cells 3A1 secreting the mAb against AFs with high affinity and specificity were prepared in this study. The LOD of this mAb to AFB_1_ is 0.023 ng/mL, and the average recoveries in and out of batches were (90.6 ± 3.4)% and (88.9 ± 2.2)%, respectively. Then, LM-ICTS based on this mAb were established, which can effectively detect AFB_1_ and AFG_1_ within 10 min. The LOD of LM-ICTS for AFB_1_ was 1.79 ng/mL, and for AFG_1_ was 8.08 ng/mL. The LM-ICTS detection method established in this study can be effectively applied to the detection of AFB_1_ and AFG_1_ in real samples, providing a sensitive, rapid, and accurate detection method for the on-site detection of key AFs in the food and feed industries.

## 4. Materials and Methods

### 4.1. Material and Reagents

Human serum albumi (HSA), bovine serum albumin (BSA), goat anti-mouse-peroxidase conjugate (IgG-HRP), Freund’s incomplete adjuvant, and Freund’s complete adjuvant were purchased from Sigma-Aldrich (St. Louis, MO, USA). Latex microsphere was purchased from Bans laboratories, Inc. (Fishers, IN, USA). AFB_1_, AFG_1_, zearalenone (ZEN), deoxynivalenol (DON), T-2 Toxin (T-2), and aflatoxin B_2_ (AFB_2_) were bought from Aladdin Biochemical Technology (Shanghai, China). The murine yeloma cell line Sp2/0 was stocked in liquid nitrogen at our laboratory. Balb/c female mice were obtained from Fuzhou Yanxi Biotechnology Co., Ltd. (Fuzhou, China). 1 L 0.01 M PBS (pH 7.4) was prepared with 8 g NaCl, 0.2 g KCl, 1.44 g Na_2_HPO_4_, and 0.24 g KH_2_PO_4_.

### 4.2. Preparation of Complete Antigens

The preparation of the complete antigen of AFB_1_ followed the previous report with minor modifications [40]. The oximation of AFB_1_ (AFB_1_-O) was prepared first: 2 mg AFB_1_ and 4 mg CMO were dissolved in 400 µL pyridine, respectively, then, 200 µL AFB_1_ and 400 µL CMO were mixed with vortex under 25 °C in dark overnight. The conjugation of AFB_1_ and carrier proteins followed the method of EDC. The oximation product AFB_1_-O in pyridine was dried by blowing with nitrogen in the fume hood, then all AFB_1_-O was dissolved in 0.64 mL N, N- Dimethylformamide (DMF) water solution (DMF/water: 2/3, *v*/*v*). To synthesize AFB_1_-HSA, 13.8 mg EDC was mixed in the AFB_1_-O solution, and then 7 mL of 10 mg/mL HSA solution (in 0.01 M PBS, pH 7.4) was added with vortex at 25 °C for 24 h. Then, the AFB_1_-HSA solution was dialyzed in phosphate buffer (0.01 M PBS, pH 7.4) at 4 °C for 3 d. Finally, the purified AFB_1_-HSA was kept at −20 °C for further experiments. The preparation of AFB_1_-BSA followed the method used for AFB_1_-HSA synthesis.

### 4.3. Mice Immunization

The experiment for mice immunization followed previous protocol with minor modifications [41]. Six-week-old male Balb/c mice from Fuzhou Yanxi Biotechnology Co., Ltd. (Fuzhou, China) were fed well with proper food and water and kept in a clean and comfortable living environment (Temperature 22 ± 2 °C, relative humidity 50 ± 5%) during the experiment. At the first immunization, Balb/c mice were injected with 200 µg of AFB_1_-HSA in 200 µL PBS (0.01 M, pH 7.4) mixed with an equal volume of Freund’s complete adjuvant. The following immunizations were performed with 50 µg immunogen in 75 µL of PBS mixed with the same volume of Freund’s incomplete adjuvant at about 2-week intervals. After six injections, the titer of the antiserum was monitored by indirect ELISA (iELISA) with AFB_1_-BSA as the coated antigen. At the end of the study, the Balb/c mice were euthanized by cervical dislocation after inhaling isoflurane, and the minimal possible number of animals were sacrificed while all efforts were made to reduce their suffering. The animal experiments in this study were conducted strictly according to the animal welfare guidelines set by the World Organization for Animal Health, and approved by the institutional ethics committee of the Key Laboratory of Pathogenic Fungi and Mycotoxins of Fujian Province, China (KLPFM, Permit number: PFMFAFU202186).

### 4.4. Indirect ELISA (iELISA)

The iELISA was performed referring to the reported procedure with slight modifications [42]. In the wells of a micro-titer plate, 1 µg/mL AFB_1_-BSA (100 µL/well) was used as the coated antigen. After the plate was washed three times with PBST (0.01 M PBS containing 0.05% *v*/*v* Tween-20) and then blotted dry, 100 µL/well serial diluted anti-AFB_1_ antiserum in 5% PBSM (0.01 M PBS containing 5% skim milk powder) (1/500, 1/1000, 1/2000, 1/4000, 1/8000, 1/16,000, 1/32,000 and 1/64,000) was added and incubated at 37 °C for 1 h. After washing with PBST, 100 µL 5% PBSM diluted goat anti-mouse IgG-HRP conjugate was added for each well and kept at 37 °C for 1 h. After washing with PBST, substrate solution was added for 15 min, then the reaction was stopped by adding 50 µL of 2 M H_2_SO_4_, and finally the result was read at 450 nm by the micro-plate reader.

### 4.5. The Preparation of the Hybridoma Cells

The preparation of anti-AFB_1_ monoclonal antibody (mAb) followed the protocol as described [41]. When the titer of the antiserum reached over 1:8000, the splenocytes from the immunized mice were mixed with SP2/0 myeloma cells at a ratio of 10:1 in 1 mL PEG (50%, polyethylene glycol, 1450) solution to enhance cell fusion. Following the selection of the hybridomas in HAT medium (RPMI + 20% FBS/HAT) with 5% CO_2_ for about 1 week, the positive hybridomas were screened by iELISA with the supernatant of their culture media. The chromosome number of the selected positive hybridoma clone was counted under the fluorescence microscope as described [43]. The subtype of the mAb was determined with the Mouse Monoclonal Antibody Subtyping Kit following the instructions of the kit.

### 4.6. Preparation of Monoclonal Antibody

After the 10-week-old Balb/c female mice were primed by intraperitoneal injection of 500 µL paraffin oil 7 d in advance, the stable antibody-secreting hybridoma cells obtained in the above step were injected intraperitoneally into the Balb/c female mice (500 μL containing about 1 × 10^6^ cells per mouse). Approximately one week later, the ascitic fluid from the mice was collected. The mAb was harvested by affinity chromatography with protein G, and the purified mAb was further analyzed with SDS-PAGE and BCA Protein Assay Kit. iELISA was used to analyze the affinity and specificity of the purified mAb [44]. The popularly used carrier protein BSA, HSA, KLH, and OVA were tested in the specificity analysis.

### 4.7. Establishment of the Standard Curve for Detection

iELISA was performed to set up an effective protocol to detect possible aflatoxins in samples according to previous publications with minor modifications [45]. AFB_1_ was diluted by 0.01 M PBS (pH 7.4) into series concentrations (25, 12.5, 6.25, 3.125, 1.563, 0.781, 0.391, 0.195, 0.097, 0.0485, and 0.0243 ng/mL) and used as competed antigen. The results of iELISA were analyzed, and the typical calibration curve and linear portion of standard curve were presented with Origin 8.0 software and plotting (B/B0). The peanut samples (free from the contamination of aflatoxins) spiked with different concentrations (including 2, 1, 0.5, and 0.025 μg/kg) of standard aflatoxins were used to assess the accuracy and recovery rate of the established detection method.

### 4.8. Preparation of Latex Microsphere (LM) Labelled mAb

The LM-mAb conjugate (LM probe) was prepared as follows [34]: First, 20 µL of 0.5% (*w*/*v*) LM solution was mixed with 1 mL of 2-(N-Morpholino) ethanesulfonic acid (MES, 0.05 M, pH 6.5) and then centrifuged at 12,000 r/min for 10 min. The precipitate was resuspended in 1 mL of MES buffer with 0.8 mg of EDC and stirred at 160 r/min for 15 min. After that, the mixture was centrifuged again, and the precipitate was resuspended in 0.5 mL of Gly buffer (50 g/L, pH 6.0). Subsequently, the optimal amount of antibody (25 µL) was added to 1 mL of the above solution to prepare the LM probe. The mixture was stirred at 160 r/min for 2 h. After centrifugation, 1 mL of Gly buffer was added to block the unbound sites on LM for 30 min. Finally, the supernatant was removed through centrifugation and the precipitate was resuspended in 20 µL of Gly buffer and stored at 4 °C for the preparation of test strips.

### 4.9. Construction of Testing Strip

The core component of an immunochromatographic test strip (ICTS) is a chromatographic strip made of absorbent material, which usually includes a conjugate pad, a sample pad, an absorbent pad, a test line (T line), and a control line (C line). When the sample to be tested is added to the sample pad, it will move along the strip towards the absorbent pad by capillary action, initiating the entire detection process. Since the number of specific antibodies fixed on the T line is limited, the analyte in the sample will compete with the labeled antigen for binding to the specific antibodies on the T line. If the concentration of the analyte in the sample is high, the amount of labeled antigen bound to the specific antibodies on the T line will decrease, resulting in a lighter or even no coloration of the T line. Conversely, if the concentration of the analyte in the sample is low, the T line will show a deeper color. The control line (C line) is used to verify whether the detection process has been carried out normally.

To rapidly and sensitively detect AFB_1_ and AFG_1_, LM-ICTS were constructed as follows: AFB_1_-BSA was used as the coating antigen and applied as a test line (T line) on the nitrocellulose (NC) membrane. Goat anti-mouse IgG was applied as a control line (C line) on the NC membrane. After incubation at 37 °C for 30 min, the test strips were cut into pieces measuring 0.2 cm in width and 6 cm in length for later use. LM-mAb was immobilized on the conjugate pad. Subsequently, the sample pad, conjugate pad, and absorbent pad were sequentially attached to the NC membrane to construct the LM-ICTS.

### 4.10. Specificity and Sensitivity of the LM-ICTS

To evaluate the specificity of the developed LM-ICTS, aflatoxin B_1_ (AFB_1_), aflatoxin B_2_ (AFB_2_), aflatoxin G_1_ (AFG_1_), zearalenone (ZEN), deoxynivalenol (DON), and T-2 toxin were tested at a concentration of 100 ng/mL (in 0.01 M PBS, pH 7.4), with PBS used as the control. The control line (C line) and test line (T line) were visually observed, and the optical intensity was recorded using the immunochromatographic analyzer C10066-10 (HAMAMATSU, Hamamatsu, Japan). The sensitivity of the proposed LM-ICTS for AFB_1_ and AFG_1_ detection was evaluated using different concentrations of AFB_1_ (1000, 500, 250, 125, 62.5, 31.25, 15.63, 7.81, 3.91, 1.95, 0.98, 0.49 ng/mL) and AFG_1_ (250, 125, 62.5, 31.25, 15.63, 7.81, 3.91, 1.95 ng/mL) with PBS as the control. The results of T/C were analyzed, and the typical calibration curve and linear portion of standard curve were presented with Origin 8.0 software and plotting (B/B0).

### 4.11. Actual Sample Testing

In order to verify the practical application effect of the LM-ICTS, peanuts, sunflower seeds, walnuts, wheat and millet were randomly purchased from a supermarket. An amount of 5 g of each sample was crushed and placed into a 50 mL centrifuge tube (in spiked samples assay, the AFB_1_ with corresponding concentration was added and mixed thoroughly after this step), then extracted with 10 mL 70% methanol. The mixture was shaken for 5 min and centrifuged at 4000 r/min for 10 min, and the supernatant was filtered through a 0.22 µm filter membrane. Then, 0.5 mL of the supernatant was mixed with 4.5 mL of PBS as the experimental group, and 100 µL of the above filtered sample extract was dropped onto the sample pad. After 10 min of reaction, the detection results were analyzed by the naked eye and quantitatively analyzed using the C10066-10 immunochromatographic analyzer.

### 4.12. Data Analysis

All the experimental data were processed and analyzed by ORIGIN8.0 (OriginLab, Northampton, MA, USA). Curve fitting and variance analysis was conducted to describe the relationship between variables. The C-line and T-line were observed by naked eyes, the signal intensity was recorded by immunochromato reader C10066-10 (HAMAMATSU, Hamamatsu, Japan), and regression analysis of the signal data was performed according to four-parameter logistic fitting with [(T/C)/(T0/C0)] as the dependent variable [46]. All analyses were performed in triplicate.

## Figures and Tables

**Table 1:** Recovery determination by spiking different concentrations of AFB_1_ into peanut samples.

Spiked Level (μg/kg)	Intra-Assay				Inter-Assay			
	*n*	Measured (μg/kg)	Recovery (%)	CV (%)	*n*	Measured (μg/kg)	Recovery (%)	CV (%)
2	3	1.8 ± 0.1	91.0 ± 3.1	3.5	3	1.8 ± 0.0	90.7 ± 0.5	0.5
1	3	1.0 ± 0.0	95.8 ± 2.7	2.8	3	0.9 ± 0.1	91.3 ± 6.4	7.0
0.5	3	0.4 ± 0.0	85.7 ± 4.9	5.9	3	0.4 ± 0.0	84.9 ± 0.2	0.2
0.3	3	0.2 ± 0.0	90.0 ± 2.8	3.1	3	0.2 ± 0.0	88.7 ± 1.7	1.8
Average			90.6 ± 3.4	3.8			88.9 ± 2.2	2.4

**Table 2:** Recovery determination of LM-ICTS by spiking different concentrations of AFB_1_ into food samples.

Sample	Spiked Level (μg/kg)	Intra-Assay				Inter-Assay			
		*n*	Measured (μg/kg)	Recovery (%)	CV (%)	*n*	Measured (μg/kg)	Recovery (%)	CV (%)
Peanuts	10	3	9.6 ± 0.8	96.6 ± 8.3	8.6	3	10.0 ± 0.9	102.0 ± 9.6	9.4
	20	3	20.1 ± 0.4	100.7 ± 2.1	2.1	3	19.0 ± 1.3	96.3 ± 6.5	6.7
	30	3	29.4 ± 0.7	98.1 ± 2.4	2.5	3	23.7 ± 2.0	79.6 ± 6.6	8.3
	Average			98.4 ± 4.3	4.4			92.6 ± 7.6	8.1
Sunflower seeds	10	3	9.3 ± 1.3	101.6 ± 13.8	13.5	3	8.8 ± 0.6	88.4 ± 6.4	7.2
	20	3	17.5 ± 0.5	87.3 ± 2.7	3.1	3	18.1 ± 0.7	90.7 ± 3.6	4.0
	30	3	28.3 ± 0.9	94.3 ± 3.1	3.3	3	26.6 ± 0.9	88.6 ± 2.9	3.3
	Average			94.4 ± 7.2	6.6			89.2 ± 4.3	4.9

## Data Availability

The original contributions presented in this study are included in the article. Further inquiries can be directed to the corresponding author(s).
